# Integrated Metabolomics, Transcriptome and Functional Analysis Reveal Key Genes Are Involved in Tree Age-Induced Amino Acid Accumulation in *Torreya grandis* Nuts

**DOI:** 10.3390/ijms242317025

**Published:** 2023-12-01

**Authors:** Weijie Chen, Jingwei Yan, Shan Zheng, Jinwei Suo, Heqiang Lou, Lili Song, Jiasheng Wu

**Affiliations:** State Key Laboratory of Subtropical Silviculture, Zhejiang A&F University, Hangzhou 311300, China; cwj86948968@163.com (W.C.); jingweiyan@zafu.edu.cn (J.Y.); 20170030@zafu.edu.cn (H.L.)

**Keywords:** metabolomics, transcriptome, tree age, amino acids, *T. grandis* nuts, TgPK

## Abstract

*Torreya grandis* is native Chinese tree species of economic significance, renowned for its long lifespan and the rich nutritional value of its nuts. In this study, we analyzed the morphological characteristics, metabolites, associated gene expressions, and regulatory mechanism in nuts from young (10 years old) and old (1000 years old) *T. grandis* trees. We observed that the length, width, and weight of nuts from older trees were considerably greater than those from younger trees. Metabolomic analysis revealed that the concentrations of 18 amino acids and derivatives (including histidine and serine) in nuts from older trees were markedly higher than those in nuts from younger trees. Transcriptome and metabolomic correlation analysis identified 16 genes, including TgPK (pyruvate kinase), TgGAPDH (glyceraldehyde 3-phosphate dehydrogenase), and others, which exhibit higher expression levels in older trees compared to younger trees, as confirmed by qRT-PCR. These genes are associated with the biosynthesis of histidine, glutamic acid, tryptophan, and serine. Transient expression of *TgPK* in tobacco led to increased pyruvate kinase activity and amino acid content (histidine, tryptophan, and serine). Additionally, dual-luciferase assays and yeast one-hybrid results demonstrated that TgWRKY21 positively regulates T*gPK* expression by directly binding to the *TgPK* promoter. These findings not only demonstrate the nutritional differences between nuts from young and old trees but also offer fresh insights into the development of nutritional sources and functional components based on nuts from old trees, enriching our understanding of the potential benefits of utilizing nuts from older trees.

## 1. Introduction

*Torreya grandis*, a member of the Taxaceae family, is commonly referred to as Chinese Torreya or Chinese nutmeg yew [1,2,3]. This large, evergreen coniferous tree produces a drupe-like fruit containing a nut seed characterized by an appealing flavor and crisp taste. Moreover, these nuts are recognized as a rich source of nutrients (amino acids, lipids, etc.) and bioactive compounds (tocopherols, polyphenols, vitamins, etc.), offering high nutritional value and multiple health effects [4]. *T. grandis* nuts are frequently processed into food supplements aimed at supporting cardiovascular health.

In the last few years, the price of roasted *T. grandis* nuts has reached 50–70 dollars per kilogram. The high economic value of *T. grandis*, which was endemic to China, has led to plantations becoming a major source of income for local farmers in Southeastern China, particularly in the subtropical mountains of Zhejiang, Anhui, and Fujian provinces. Due to this increasing demand, both the acreage and nut production of *T. grandis* have expanded rapidly. For instance, the acreage of *T. grandis* in China increased sixfold from approximately 10,000 ha in 2000 to around 60,000 ha in 2020 [5]. Notably, *T. grandis* is a perennial plant species with an exceptionally long lifespan, capable of surviving and producing nuts for centuries or even millennia [3]. The Kuaiji mountain region in Zhejiang province, an original cultivation area for *T. grandis*, hosts the largest ancient *T. grandis* community, accounting for over 80% of China’s nut production. To date, more than 4000 *T. grandis* trees with a stand age exceeding 1000 years have been identified in the region. The annual yield of nuts from old trees is significantly higher than that of nuts from younger trees [5,6]. However, the differences in nut composition between young and old *T. grandis* trees remain unknown.

Tree age significantly influences the size and quality of fruit. For instance, the single fruit weight and skin thickness of crystal honey pomelo exhibit a downward trend as tree age increases, while the soluble sugar and vitamin C content initially rise before declining with increasing tree age [7]. In apples, fruit mass and size notably increase before the tree reaches the full fruiting stage [8]. In Newhall navel oranges, young trees have larger fruit, greater fruit weight, and thicker peel than older trees, whereas total soluble solids (TSS) display an opposite trend [9]. In Chinese chestnut, both the 10-year-old and 700-year-old groups exhibited higher levels of zinc, selenium, and amino acid compared to trees of other ages [10]. In *Ziziphus jujuba*, as the age of the tree increases, the content of reducing sugars in the fruit also gradually increases [11]. These studies suggest that the size and quality of fruit vary with tree age across different plant species. Amino acids are bioactive compounds in *T. grandis* nuts that contribute to sensory properties, such as taste, aroma, and color, in addition to their nutritional value. However, differences in amino acid or other metabolite composition between young and old *T. grandis* tree nuts remain unknown and may impact sensory and nutritive attributes.

In this study, nuts from young (10 years old) and old (1000 years old) *T. grandis* trees were collected from the primary *T. grandis* plantation in China. By comparing the metabolic differences in nuts from young and old *T. grandis* trees, we can reveal the physiological characteristics and adaptability of trees at different growth stages in terms of metabolic pathways and nutrient utilization. This contributes to understanding the intrinsic laws of *T. grandis* growth and development, providing a scientific foundation for its cultivation and management. Furthermore, acquiring in-depth insight into the quality, yield, and nutritional components of nuts from young and old *T. grandis* trees is essential for improving nut yield and quality. Elucidating the differences between nuts from young and old trees allows for the implementation of differentiated management strategies tailored to different age trees, thereby maximizing their potential in terms of yield and quality. Moreover, research on fruit trees has demonstrated the significant impact of age on fruit quality, highlighting the importance of studying long-lived nut trees, such as *T. grandis*. Therefore, filling this knowledge gap in the field will provide a more comprehensive and in-depth scientific basis for the cultivation and management of nut-bearing tree species, potentially driving the sustainable development of related industries.

## 2. Results

### 2.1. Morphological Changes in Young and Old T. grandis Nuts

**Figure 1:**
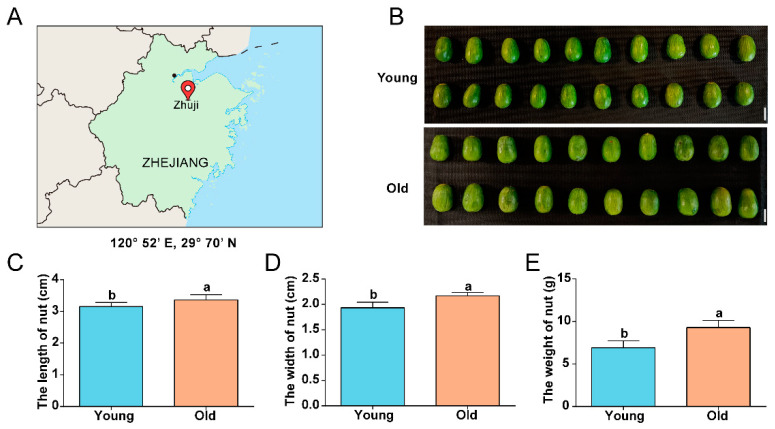
Morphological characteristics of *T. grandis* nuts. (**A**) Sample location of *T. grandis* nuts in this study. (**B**) Photographs of young and old *T. grandis* nuts. The length (**C**), width (**D**), and weight (**E**) of nuts from young and old *T grandis* trees. The scale bar in (**B**) indicates 2 cm. Error bars in (**C**–**E**) represent SD (*n* = 30). Different letters in (**C**–**E**) indicate a significant difference compared to the control (young) as determined by the unpaired Student’s *t*-test (*p* < 0.05).

To compare the differences in nuts between young and old *T. grandis* trees, we analyzed the morphological characteristics of nuts from both young and old trees. The nuts of the old tree were significantly larger than those of the young tree, with greater length and width. The weight of old tree nuts was approximately 1.33 times higher than that of young tree nuts (Figure 1). These results suggest that tree age positively affects nut morphology-related changes.

### 2.2. Metabolites Analysis in T. grandis Nuts of Young and Old Trees

**Figure 2:**
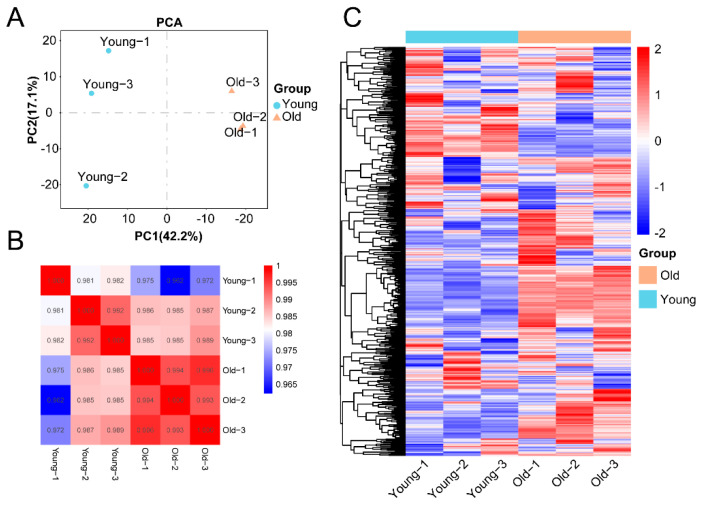
Metabolites profiling in young and old *T. grandis* nuts. (**A**) Principal component analysis (PCA) of metabolites between young and old *T. grandis* nuts. (**B**) Pearson correlation of metabolites between young and old *T. grandis* nuts. (**C**) Heatmap plot of metabolites in young and old *T. grandis* nuts.

To further understand the differences in metabolites between young and old *T. grandis* nuts, nuts from young (10 years old) and old (1000 years old) *T. grandis* trees were subjected to liquid chromatography tandem mass spectrometry (LC-MS/MS). A total of 945 annotated metabolites were identified (Table S2), encompassing 184 amino acids and derivatives, 79 carbohydrates and their derivatives, 71 organic acids and their derivatives, 69 nucleotides and their derivatives, and other metabolites. The *T. grandis* nut samples were sorted into distinct clusters, indicating substantial differences in metabolite levels between old and young trees (Figure 2A,B). Heatmap analysis revealed that the metabolites from old and young trees were segregated into two main clusters (Figure 2C). Amino acids and derivatives were more abundant in old tree nuts compared to young tree nuts, while the majority of alkaloids, nucleotides, and derivatives were relatively higher in young tree nuts, accounting for the depletion in amino acids with age (Table S2). Amino acids serve as precursors for alkaloid and nucleotides biosynthesis [12].

**Figure 3:**
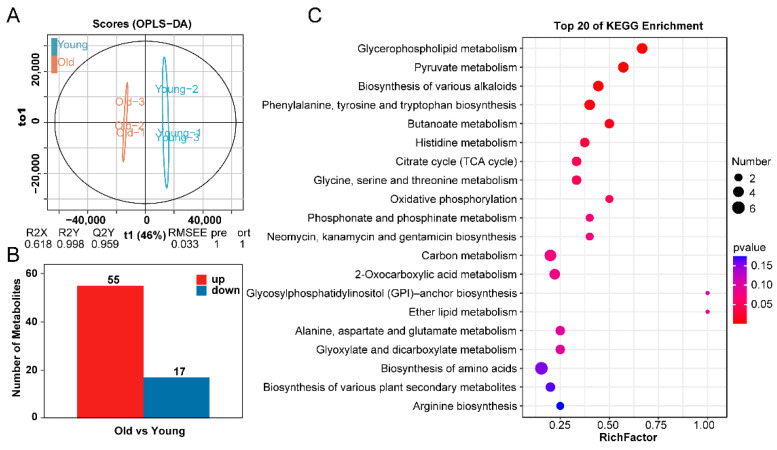
Identification of differentially expressed metabolites between young and old *T. grandis* nuts. (**A**) Differentially expressed metabolites of *T. grandis* nuts via OPLS-DA analysis. (**B**) The number of differentially expressed metabolites in *T. grandis* nuts. (**C**) KEGG enrichment analysis of differentially expressed metabolites of *T. grandis* nuts.

Differential metabolites were further examined using supervised orthogonal partial least squares discriminant analysis (OPLS-DA) to confirm the results from unsupervised principal component analysis (PCA). The results demonstrated that the predictive power of models was good, with a Q2 value of 0.959 (Figure 3A). A total of 72 differential metabolites, including 55 up-regulated metabolites and 17 down-regulated metabolites, were identified in old *T. grandis* nuts compared with young tree nuts (Figure 3B). We then examined the enriched KEGG pathways for the targeted metabolites with differential accumulation in *T. grandis* nuts. The top 20 enriched pathways are displayed in Figure 3C. All projected target metabolites were enriched in 61 KEGG pathways (Table S3). The KEGG pathways with the greatest enrichment included glycerophospholipid metabolism (ko00564), pyruvate metabolism (ko00620), biosynthesis of various alkaloids (ko00996), phenylalanine, tyrosine, tryptophan biosynthesis (ko00400), and butanoate metabolism (ko00650). These metabolic pathways are involved in multiple aspects of plant growth and development, encompassing the formation and maintenance of cell structures, energy metabolism, and the synthesis of secondary metabolites. They play crucial regulatory roles in the growth, development, and environmental adaptation of plants.

### 2.3. Analysis of Amino Acids Metabolites in T. grandis Nuts

**Figure 4:**
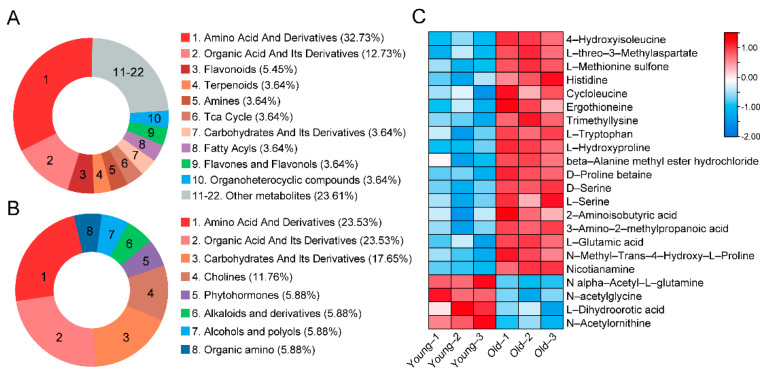
Identification of differentially expressed amino acids between young and old *T. grandis* nuts. (**A**) The proportion of up–regulated metabolite categories. (**B**) The proportion of down–regulated metabolite categories. (**C**) Heatmap of differential metabolites in amino acid and derivatives between young and old *T. grandis* nuts.

Given that 22 amino acids accounted for 30.6% of the differential peaks detected in the nut metabolome from old and young trees (Table S4), we conducted a detailed analysis of these amino acids. Among the 22 detected amino acids, 18 exhibited an increase in old tree nuts, representing 32.7% of the total up-regulated metabolites (Figure 4A) and 23.5% of the down-regulated metabolites (Figure 4B). Relative changes in amino acid and nitrogenous compounds between young and old *T. grandis* nuts included L−glutamic acid, 4−hydroxyisoleucine, L−threo−3−methylaspartate, histidine, and others (Figure 4C).

### 2.4. Identification of Genes Potentially Involved in Amino Acid Biosynthesis through Transcriptome Analysis

**Figure 5:**
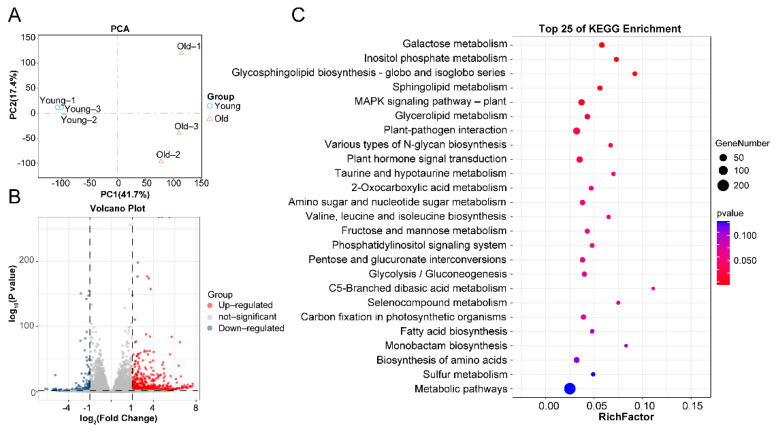
Differential expressed genes between young and old *T. grandis* nuts. (**A**) Principal component analysis (PCA) of gene expression dataset in *T. grandis* nuts. (**B**) Volcano plots for DEGs in *T. grandis* nuts. (**C**) KEGG classification of DEGs in *T. grandis* nuts.

To further explore the potential molecular mechanisms underlying changes in nut metabolism, we used nuts from young and old trees for RNA-seq analysis. cDNA libraries were submitted for high-throughput parallel sequencing to better understand amino acid biosynthesis at the transcriptional level. After removing adaptor sequences and low-quality reads, total clean reads and clean bases were produced, ranging from 39.62 to 49.32 million (M) and 5.94 to 7.03 Gb, respectively. GC proportions varied between 43.57% and 44.59%. Q20 and Q30 values were also obtained, ranging from 97–98% and 93.5–94.3%, respectively, and used to evaluate the quality of the sequencing bases (Table S5). Additionally, the transcriptome dataset was subjected to PCA to ascertain whether the same pattern in nut segregation could be observed at the transcription level (Figure 5A) and the underlying gene expression pattern. To identify differentially expressed genes (DEGs), the following criteria had to be satisfied: expression difference fold |log2FoldChange| ≥1, significant *p*-value < 0.05. Compared with young tree nuts, a total of 2321 DEGs, comprising 1400 up-regulated and 921 down-regulated genes, were found in old tree nuts (Figure 5B and Table S6). We further analyzed the enriched KEGG pathways of the DEGs. The up-regulated DEGs were enriched in 122 KEGG pathways. Interestingly, the ‘biosynthesis of amino acids’ was notably enriched (Figure 5C). A total of 27 genes were enriched in this pathway, including 19 up-regulated genes and eight down-regulated genes in old tree nuts (Table S7).

### 2.5. Identification of Key Genes Related to Amino Acid Biosynthesis in Nuts

**Figure 6:**
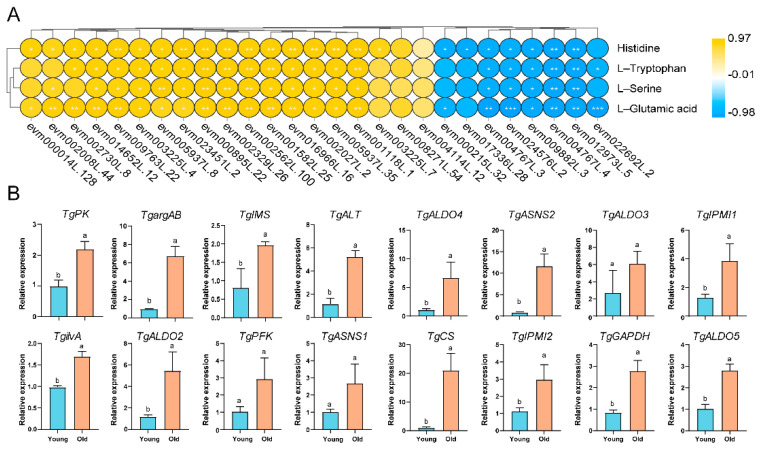
DEGs related to key amino acid biosynthesis in young and old *T. grandis* nuts. (**A**) Correlation analysis of key amino acids and DEGs involved in amino acids biosynthesis. (**B**) Relative expression levels of 16 genes involved in key amino acids biosynthesis between young and old *T. grandis* nuts via qRT-PCR. Error bars in (**B**) indicate SD (*n* = 3). Different letters in (**B**) indicate a significant difference compared to the control (young) as determined by the unpaired Student’s *t*-test (*p* < 0.05).

To further explore the potential molecular mechanisms underlying amino acid regulation in nuts, 27 genes and four amino acids (histidine, L−glutamic acid, L−tryptophan, and L−serine) found to be abundant in old tree nuts were modeled together in a co-expression network based on Pearson correlation analysis (Figure 6A). The results showed that 1 TgPK (pyruvate kinase), 1 TgGAPDH (glyceraldehyde 3-phosphate dehydrogenase), 1 TgALT (alanine transaminase), 1 TgPFK (6-phosphofructokinase 1), 4 TgALDOs (fructose-bisphosphate aldolase), 1 TgIMS (2-isopropylmalate synthase), 2 TgIPMIs (3-isopropylmalate/(R)-2-methylmalate dehydratase small subunit), 1 TgilvA (threonine dehydratase), 3 TgASNSs (asparagine synthase), and 1 TgargAB (amino-acid N-acetyltransferase) were positively and strongly associated with at least 2 amino acids (Figure 6A, Figure S1 and Table S7). A qRT-PCR assay was further used to confirm the expression patterns of these genes for validation (Figure 6B). These results suggest that these genes significantly contribute to amino acid biosynthesis in old *T. grandis* nuts.

### 2.6. Sequence Alignment and Phylogenetic Analysis of TgPK

**Figure 7:**
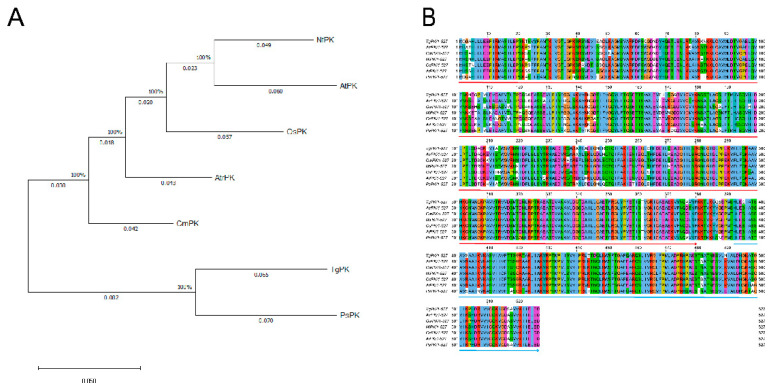
Sequence alignment and phylogenetic analysis of TgPK. (**A**) Phylogenetic analysis of TgPK and closely related PK protein in other species using the MEGA X program. NtPK (*Nicotiana tomentosiformis*, XP_009627228.1), AtPK (*Arabidopsis thaliana*, NP_566976.1), OsPK (*Oryza sativa*, NP_001391374.1), AtrPK (*Amborella trichopoda*, XP_006851775.1), CmPK (*Cinnamomum micranthum* RWR82390.1), PsPK (*Picea sitchensis*, ABK24322.1). (**B**) Alignment of the TgPK with closely related PK protein in other species using Jalview 2.11.2.6. The putative pyruvate kinase domains are marked by a red and blue arrow, respectively. Pfam: PK (PF00224) and PK_C (PF02887).

Pyruvate kinase (PK) is crucial for ATP production and influences fatty acid and amino acid synthesis via the tricarboxylic acid cycle. Consequently, we focused on the important PK gene based on the differential gene analysis of the amino acid synthesis pathway mentioned above. First, to investigate the amino acid sequence homology between TgPK and other PKs, a phylogenetic analysis was generated using the maximum likelihood method. TgPK was closely associated with PsPK from *Picea sitchensis*, and these two PKs formed an independent branch, distinguishing them from other angiosperm PKs (Figure 7A). Sequence alignment indicated that TgPK exhibited high similarity with other PK proteins. TgPK contained PK (PF00224) and PK_C (PF02887) conserved domains, which were also present in other PK proteins (Figure 7B).

### 2.7. TgPK Regulates the Accumulation of Histidine, Tryptophan, and Serine

**Figure 8:**
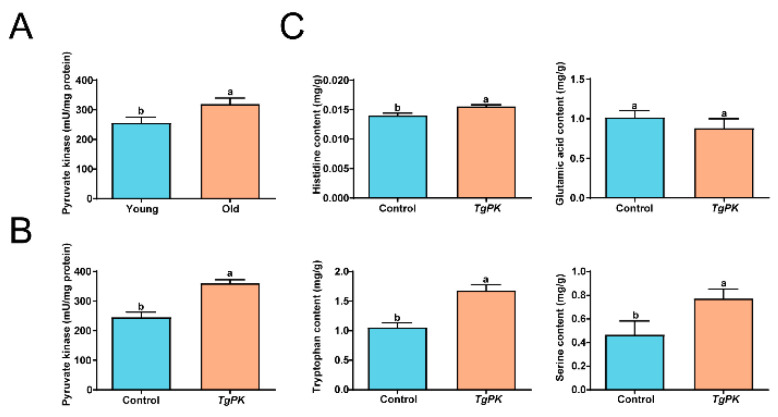
TgPK regulates the accumulation of histidine, tryptophan, and serine. (**A**) Pyruvate kinase activity in nuts from young and old trees. (**B**) Pyruvate kinase activity in transiently overexpressed tobacco leaves. (**C**) Amino acid content (histidine, glutamic acid, tryptophan, and serine) in transiently overexpressed tobacco leaves. Error bars in (**A**–**C**) indicate SD (*n* = 3). Different letters in (**A**–**C**) indicate a significant difference compared to the control (young) as determined by the unpaired Student’s *t*-test (*p* < 0.05).

To confirm the role of TgPK in amino acid synthesis, we examined the activity of PK in nuts from young and old *T. grandis* trees. We found that the activity of pyruvate kinase in old *T. grandis* tree nuts was significantly higher than that in young tree nuts (Figure 8A). To further confirm whether TgPK could affect amino acid content, *TgPK-GFP* or *GFP* driven by the *35S* promoter was transiently expressed in tobacco leaves. We noted that PK activity was higher in leaves transiently expressing *TgPK-GFP* than in leaves expressing *GFP* (Figure 8B). The amino acid contents, including histidine, tryptophan, and serine, were also considerably greater in leaves transiently expressing *TgPK-GFP*, consistent with the PK activity (Figure 8C). These results suggest that TgPK participates in amino acid (histidine, tryptophan, and serine) synthesis by increasing PK activity in tobacco leaves.

### 2.8. TgWRKY21 Regulates the TgPK Expression via Direct Binding to the TgPK Promoter

**Figure 9:**
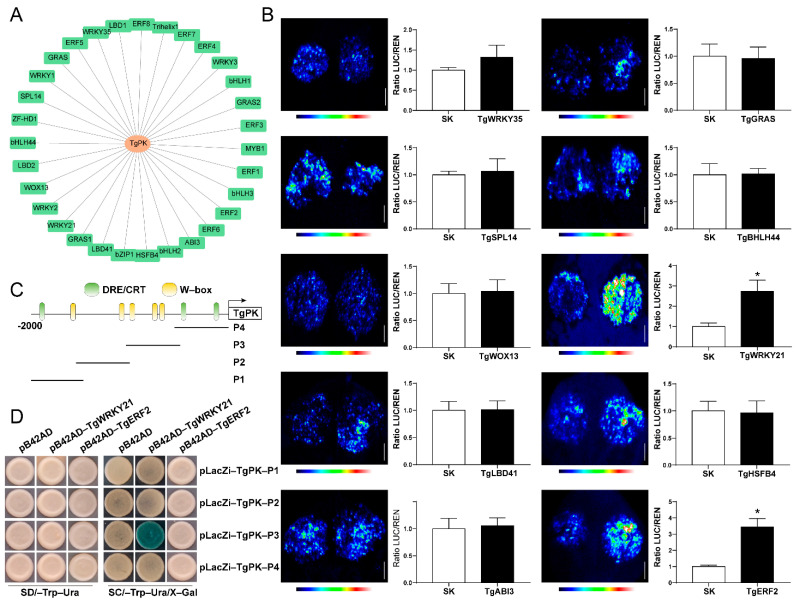
TgWRKY21 regulates the *TgPK* expression via direct binding to the *TgPK* promoter. (**A**) Pearson correlation analysis between *TgPK* expression levels and top transcription factors (22) from transcriptome data. (**B**) Effect of transcription factors on LUC activity driven by *TgPK* promoter via dual-luciferase analysis. Scale bar corresponds to 1 cm. (**C**) Potential motifs recognized by WRKY and ERF proteins in *TgPK* promoter. (**D**) TgWRKY21 directly binds to the *TgPK* promoter in yeast. Yeast EGY48 strain co-transformed with fragments of *TgPK* promoter and pB42AD-TgWRKY21 or empty pB42AD vector were grown on a synthetic dropout/-Trp-Ura/Gal/Raf/X-Gal (80 μg/mL) plate for three days. Error bars in (**B**) indicate SD (*n* = 3). (**B**) indicates a significant difference compared to the control (young) as determined by the unpaired Student’s *t*-test (* *p* < 0.05).

Since *TgPK* transcript levels varied with tree age, we hypothesized that transcriptional regulation played a critical role in this process. Accordingly, we initially conducted a co-expression analysis to identify major transcription factors potentially regulating *TgPK* expression. As shown in Figure 9A, 22 transcription factors with a Pearson correlation coefficient of less than 0.95 were substantially associated with *TgPK*. The top 10 transcription factors, such as WRKY, ERF, WOX, and LBD, were chosen for dual-luciferase assays to identify potential regulators that were involved in regulating *TgPK* expression. We found that only TgERF2 and TgWRKY21 enhanced the activity of LUC driven by the *TgPK* promoter, while other transcription factors had no significant effect on this activity (Figure 9B). Subsequently, we identified five W-box ((T)TGAC(C/T)) and three DRE/CRT (CCGAC) motifs recognized by WRKY and ERF proteins, respectively (Figure 9C). The *TgPK* promoter was divided into four fragments (P1–P4) based on cis-element distribution (Figure 9C). These four promoter fragments of *TgPK* were constructed into the pLacZi vector, while *TgERF2* and *TgWRKY21* were inserted into the pB42AD vector. Yeast one-hybrid assays revealed that yeast EGY48 transformed with *TgWRKY21,* and P3 fragment displayed blue, indicating that TgWRKY21 is directly bound to the *TgPK* promoter in yeast. Taken together, these results suggest that TgWRKY21 positively regulates *TgPK* expression by directly binding to the promoter of *TgPK*.

## 3. Discussion

In this study, we analyzed the morphological characteristics, metabolites, associated gene expressions and regulatory mechanisms in nuts from young and old *T. grandis* trees. The results demonstrated that the weight, length, and width of nuts from old trees were considerably greater than those from younger trees, indicating a positive correlation between tree age and nut morphology (Figure 1). Similar trends have been observed in other fruit species. For example, in pummelo, fruit weight, peel weights, diameter, and circumference all increase with tree age [13]. Khalid et al. [14] found that seed mass in young ‘Kinnow’ mandarin trees (*Citrus nobilis* Lour × *Citrus deliciosa* Tenora) was poor, possibly due to low macronutrient (P and Ca) and micronutrient (Cu, Mn, Fe, and Zn) levels [14]. Older *T. grandis* trees, with well-developed root systems, exhibit greater nutrient adsorption capabilities, leading to higher nutrient accumulation [3]. Additionally, older *T. grandis* trees, being taller and having more branches, might possess enhanced photosynthesis, resulting in increased biomass production. Furthermore, during the long period of growth, older trees generally have a more balanced and stable nutrient allocation, which can better fulfill the growth requirements of the nuts [15]. Lastly, due to years of adaptation and growth, older trees have a stronger ability to adapt to the environment and are better able to resist external stresses, such as pests and diseases, thereby contributing to stable and increased fruit yield [16]. These factors could contribute to the larger nut size in older *T. grandis*. Genes involved in amino acid biosynthesis are up-regulated in old trees because amino acids play a crucial role in various biological processes such as growth, tissue repair, and stress response [17]. Consequently, the upregulation of genes involved in amino acid biosynthesis in old trees might help maintain protein synthesis and metabolism, protect cells from stress and damage, and contribute to the synthesis of essential signaling molecules. This enhanced process can facilitate nutrient optimization and resource utilization in plants, ultimately promoting rapid growth and development of nuts, potentially leading to increased yield and larger nut size.

Using LC-MS/MS-based widely targeted metabolomics analysis, we identified 945 metabolites in nuts from young and old trees. The abundance of amino acids contributes to the nuts’ pleasant flavor and high quality [18]. A total of 72 differential metabolites were identified in old *T. grandis* nuts compared to young tree nuts. The KEGG pathways with the greatest enrichment included glycerophospholipid metabolism, pyruvate metabolism, and biosynthesis of various alkaloids. These differences may stem from the varying adaptability of *T. grandis* trees at different growth stages to environmental, nutritional, and growth conditions. These variances likely reflect the tree’s need to dynamically regulate its metabolic activities in response to changes in the external environment during different growth stages. The proportion of amino acids and derivatives is the highest among these. A total of 18 amino acids and derivatives, including histidine, glutamic acid, tryptophan, and serine, were found in higher concentrations in nuts from old trees (Figure 4). Interestingly, in Chinese chestnut trees, both young (10 years old) and old (700 years old) trees exhibit higher amino acid content compared with other stages [10]. Glutamic acid, which has a pronounced umami taste, may provide a pleasant savory taste, suggesting that older *T. grandis* nuts could have a superior taste [19]. Additionally, as an indispensable amino acid obtained through dietary protein, tryptophan plays crucial roles in mood, behavior, and immune responses [20,21]. Histidine supplementation was found to suppress food intake and fat accumulation in rats [22]. These findings imply that nuts from older *T. grandis* trees may have higher food and medical value.

Numerous genes, such as 3-deoxy-d-arabino-heptulosonate-7-phosphate synthase (DAHP), anthranilate synthase (ASA), citrate synthase (CITS), have been identified as being involved in amino acid biosynthesis. Our previous study identified four genes, including *TgDAHP* and *TgASA*, as potentially involved in the biosynthesis of amino acids in young *T. grandis* nuts. In the current study, we further identified that 16 genes (*TgPK*, *TgGAPDH*, *TgALT*, etc), which were detected at substantially higher levels in old tree nuts and associated with the biosynthesis of histidine, glutamic acid, tryptophan, and serine based on comparative transcriptome and metabolomics analyses. Understanding the specific genetic factors contributing to the increased levels of these essential amino acids can potentially inform targeted strategies aimed at enhancing the nutritional quality and yield of nuts. The central component of the amino acid synthesis pathway involves the conversion of three-carbon compounds from glyceraldehyde-3P to pyruvate. As the final enzyme of the central component, PK catalyzes the conversion of phosphoenolpyruvate and ADP to pyruvate. By regulating PK activity, the flow of the glycolysis pathway could be affected, leading to alterations in the levels of ATP and NADH within the cells, thus influencing the energy supply and raw material provision for the amino acid synthesis pathway. Therefore, PK plays a crucial role in regulating the balance of amino acid metabolism. Mutations of *PKC3* or *PKC4* in *Arabidopsis* resulted in a decrease in the content of some downstream amino acids [23]. Our study provides novel evidence that *TgPK* may be a key gene responsible for the higher quantities of tryptophan, histidine, and serine found in old tree nuts compared with young tree nuts based on the following reasons: (i) *TgPK* expression was higher in old tree nuts than in young tree nuts based on RNA-seq and qRT-PCR detection results; (ii) transient overexpression of *TgPK* in tobacco leaves significantly increased PK enzyme activity and amino acid content in the leaves. Transcriptional regulation plays a crucial role in amino acid synthesis during plant seed development and maturation. DkWRKY3 and DkWRKY15 can promote the transcription of the PK gene *DkPK1*, positively influencing the natural deastringency of persimmon fruit [24]. In this study, we found that TgWRKY21 could directly bind to the *TgPK* promoter and regulate its expression. These new genes-related amino acid biosynthesis identified in this study further provide a basis for the regulation of these key amino acids and future biotechnological attempts to improve production yield in *T. grandis* nuts.

## 4. Material and Methods

### 4.1. Plant Material and Morphological Measurement

*T. grandis* cv. Merrillii grafting trees “Xifei” located in Zhaojia Town, Zhuji City, China (120°52′ E, 29°70′ N) were utilized in this study. Seeds were obtained from young (10 years old) and old (1000 years old) *T. grandis* trees. In September 2022, 30 samples in each tree age were collected from six trees, which was the mature stage of *T. grandis* nuts (17 months after flowering). After removing the external scales of *T. grandis* nuts, they were frozen in liquid nitrogen and stored at −80 °C for subsequent metabolomics and transcriptome analysis. Electronic scales and vernier calipers were utilized to measure the weight, length, and width of *T. grandis* nuts (*n* = 30 from 6 trees).

### 4.2. Metabolites Extraction and Analysis

Metabolite extraction and analysis were conducted by Novogene Co. Ltd. (Tianjin, China). The *T. grandis* nuts were processed according to manufacturer instructions. Briefly, the sample (100 mg) was ground using liquid nitrogen separately. The resulting homogenate was suspended in pre-cooled 80% methanol using a vortex mixer for 20 min. A portion of the supernatant was then diluted with LC-MS grade water to obtain a final concentration containing 53% methanol. The resulting solution was transferred to a fresh Eppendorf tube and centrifuged again at 15,000× *g*, 4 °C for another 20 min. Finally, the supernatant was injected into the LC-MS/MS system for analysis. LC-MS/MS employed a QTRAP^®^ 6500+ mass spectrometer (SCIEX, Redwood City, CA, USA) and an ExionLC^TM^ AD system (SCIEX, Redwood City, CA, USA). Chromatographic separation was performed using a Xselect HSS T3 (2.1 × 150 mm, 2.5 μm) (Waters, Milford, MA, USA). Eluent A (0.1% formic acid in water) and eluent B (0.1% formic acid in acetonitrile) followed this solvent gradient: 2% B, 2 min; 2–100% B, 15 min; 100% B, 17 min; 100–2% B, 17 min; 2% B, 20 min. QTRAP^®^ 6500+ mass spectrometer was operated in positive polarity mode with 35 psi Curtain Gas pressure, Medium Collision Gas, 5500 V IonSpray Voltage, 550 °C, and 1:60 for both Ion Source Gas 1 and Ion Source Gas 2. In negative polarity mode, the settings were the same except for −4500 V IonSpray Voltage. MS data were acquired using Multiple Reaction Monitoring (MRM), and metabolite quantification was performed using the Q3. To identify the metabolite, Q1, Q3, RT (retention time), DP (declustering potential), and CE (collision energy) were employed. The SCIEX OS Version 1.4 (SCIEX, Redwood City, CA, USA) was used to process the LC-MS/MS data files and for data visualization. The main parameters were set with a minimum peak height of 500, signal/noise ratio of 5, and Gaussian smooth width of 1. The relative content of each peak is represented by its area.

The KEGG (https://www.genome.jp/kegg/pathway.html, accessed on 23 October 2022) and HMDB (https://hmdb.ca/metabolites, accessed on 23 October 2022) databases were used to annotate the metabolites. To identify differential metabolites, orthogonal partial least squares discriminant analysis (OPLS-DA) model and variable importance in projection (VIP) values were utilized. The screening criteria were used for *p*-value ≤ 0.5 and VIP ≥ 1. For heat maps, the data were normalized using z-scores of the intensity areas of differential metabolites. The correlation between differential metabolites was analyzed by Pearson. Statistically significant correlations between differential metabolites were calculated using the R language. A *p*-value of less than 0.05 was considered statistically significant, and correlation plots were generated using OmicShare (https://www.omicshare.com/, accessed on 1 June 2023). The functions of these metabolites and their associated metabolic pathways were studied using the KEGG database.

### 4.3. RNA Isolation and Qualification

Total RNA was extracted from young and old *T. grandis* nuts using TianGen reagent (Beijing, China) as described by Yan et al. [25]. Briefly, about 100 mg of tissue was placed in liquid nitrogen, thoroughly ground with a mortar and pestle, 500 μL of Buffer SL was added (ensuring β-ME addition before use), and vortexed vigorously. After the lysate was centrifuged, transferred, and had ethanol added before being centrifuged again for discarding the flow-through, buffer RW1 was added to spin column; following this, the DNase I working solution was introduced and left for 15 min at room temperature, and finally, after multiple steps of centrifugation and buffer addition, the spin column membrane was dried, followed by the addition of RNase-Free water to elute the RNA, which should be stored at −70 °C for later use. The purity, concentration, and integrity of RNA samples were assessed using a NanoDrop 2000 spectrophotometer.

### 4.4. Transcriptome Sequencing

The cDNA library was constructed from high-quality RNA, and the resulting 150 bp paired-end reads were sequenced using the Illumina NovaSeq 6000 platform [26]. Raw data in fastq format were processed using custom perl scripts to obtain clean reads by removing adapter-containing, ploy-N-containing, and low-quality reads while also calculating Q20 and Q30 by FastQC [27]. Reference genome and gene model annotation files were downloaded from Figshare database (https://doi.org/10.6084/m9.figshare.21089869, accessed on 23 October 2022). Clean reads were mapped to the reference genome of *T. grandis* [28] using HISAT 2 v2.0.5 with default parameters [29], and the featureCounts (v1.5.0) software was utilized to count the mapped reads for each gene, followed by the calculation of FPKM (Fragments Per Kilobase of transcript per Million mapped reads) for each gene based on its length and mapped read count [30]. The samples underwent cluster analysis using principal component analysis (PCA) and Pearson correlation analysis. DESeq R (4.0.4) software identified differentially expressed genes (DEGs) in each sample group based on *p*-value < 0.05 and fold change ≥ 2 changes [31]. The acquired unigenes were blasted and annotated against NCBI non-redundant protein sequences (Nr), Swiss-Prot databases, evolutionary genealogy of genes with non-supervised orthologous groups (eggNOG), Kyoto encyclopedia of genes and genome (KEGG), and Gene ontology (GO).

### 4.5. Real-Time RT-PCR Analysis

Total purified RNA was converted to cDNA using the cDNA reverse transcription kit (RR036B, PrimeScript^TM^ RT Master Mix, Takara, Kusatsu, Shiga, Japan). Following dilution, Vazyme’s ChamQ SYBR qPCR Master Mix kit (Q711, Vazyme, Nanjing, China) and the CFX96 Touch^TM^ Thermal Cycler device were used to perform qRT-PCR (C1000, Bio-Rad, Hercules, CA, USA). The housekeeping gene *TgActin* served as a reference in the 2^−ΔΔCT^ technique to normalize transcript levels [32]. All primer sequences can be found in Table S1.

### 4.6. Comparative Analysis

The unpaired Student’s *t*-test was used to compare nut morphology, gene expression, and PK enzyme activity between young and old trees, as well as to analyze the differences in amino acid expression levels between overexpressed tobacco leaves and controls. The expression levels of amino acids and the FPKM values of differentially expressed genes were used for Pearson correlation analysis. When *p* < 0.05, it was marked as a significant difference. Meanwhile, *TgPK* and differentially expressed transcription factors were also subjected to Pearson correlation analysis, and when the correlation coefficient was greater than 0.95, it was retained and used to construct a co-expression network, which was visualized using the CYTOSCAPE software 3.8.2 [33].

### 4.7. Dual-Luciferase Assay

While the full-length coding sequences of screened transcription factors were cloned into pGreen II 62-SK vector to construct the effectors, the promoter sequence of *TgPK* (2000 bp) was cloned into pGreen II 800-LUC vector. *Agrobacterium tumefaciens* GV3101 carrying the produced effector and reporter plasmids were co-transformed into tobacco. To assess the activity of LUC and REN luciferase, a dual-luciferase assay kit (Promega) was employed.

### 4.8. Yeast One-Hybrid (Y1H) Assay

The promoter sequence containing the core cis element of *TgPK* was split into four segments and inserted into the vector pLacZi, whereas the full-length coding sequences of *TgWRKY21* and *TgERF2* were inserted into the vector pB42AD. The constructs were co-transformed into the EGY48 yeast strain, and then, the strain was cultured on a dropout/-Trp-Ura/Gal/Raf/X-Gal plate for 3 days.

### 4.9. Transient Expression of TgPK in Tobacco Leaves

The *Agrobacterium tumefaciens* strain GV3101 carrying *TgPK-GFP* or *GFP* was transformed into the four-week-old *Nicotiana benthamiana* leaves. After culturing for 3 days, the leaves that transiently expressed *TgPK-GFP* or *GFP* were collected to measure the pyruvate kinase activity and amino acid content.

### 4.10. Analysis of Pyruvate Kinase Activity

The harvested tobacco leaves and *T. grandis* nuts were ground with liquid nitrogen, homogenized with PBS buffer, and vortexed thoroughly. After centrifuging twice, the supernatant was used to measure pyruvate kinase activity using the Plant Pyruvate Kinase kit (PK–1–Y, Comin, Suzhou, China).

### 4.11. Analysis of Amino Acid Content

Sample (0.5 g) was mixed with 3 mL of extraction buffer (20% ethanol solution containing 0.001 mol/L HCl) and subjected to sonication at 4 °C for 30 min for amino acid extraction. Following centrifugation at 12,000× *g* for 5 min, the supernatant was collected. After filtering with an aqueous membrane (0.22 µm pore size), the amino acid concentration was determined using HPLC-MS/MS (AGILENT 1260 and AB 4000, Santa Clara, CA, USA). The HPLC conditions were as follows: Column, Information-HILICZ (2.7 µm, 3.0 mm × 100 mm); mobile phase, 75% acetonitrile (*V/V*) (solvent A) and 0.1 mol/L sodium acetate (solvent B); flow rate, 0.3 mL/min; column temperature, 35 °C; injection volume, 1 µL.

## 5. Conclusions

In this study, we analyzed the morphological characteristics, metabolites, and the related gene expressions in young and old *T. grandis* nuts. The results demonstrated that the length, width and weight of nuts from old trees (1000 years old) were significantly greater than those from young trees (10 years old). A total of 18 amino acids and derivatives, including histidine, glutamic acid, tryptophan, and serine, were present in higher quantities in old tree nuts compared to young tree nuts. Moreover, 16 genes, expressed at higher levels in old tree nuts, were associated with the biosynthesis of histidine, glutamic acid, tryptophan, and serine according to transcriptome and correlation analysis. Transient expression of *TgPK* in tobacco led to increased pyruvate kinase activity and amino acid (tryptophan, histidine, and serine) content. Additionally, TgWRKY21 was found to positively regulate *TgPK* expression by directly binding to the *TgPK* promoter. These findings not only provide information on the nutritional differences between nuts from young and old trees but also offer novel insight for the development of valuable nutritional sources and functional ingredients based on old *T. grandis* nuts. Future research should include sensory analysis and biological assays based on these promising findings derived from metabolomics analysis.

## Data Availability

The data presented in this study are available on request from the corresponding author.

## Supplementary Materials

The following supporting information can be downloaded at: https://www.mdpi.com/article/10.3390/ijms242317025/s1.
