# Supplementary figures and images for: Integrated Metabolomics, Transcriptome and Functional Analysis Reveal Key Genes Are Involved in Tree Age-Induced Amino Acid Accumulation in Torreya grandis Nuts
Source: Int J Mol Sci. 2023 Dec 1;24(23):17025. doi: 10.3390/ijms242317025 (PMC10706915; doi:10.3390/ijms242317025)

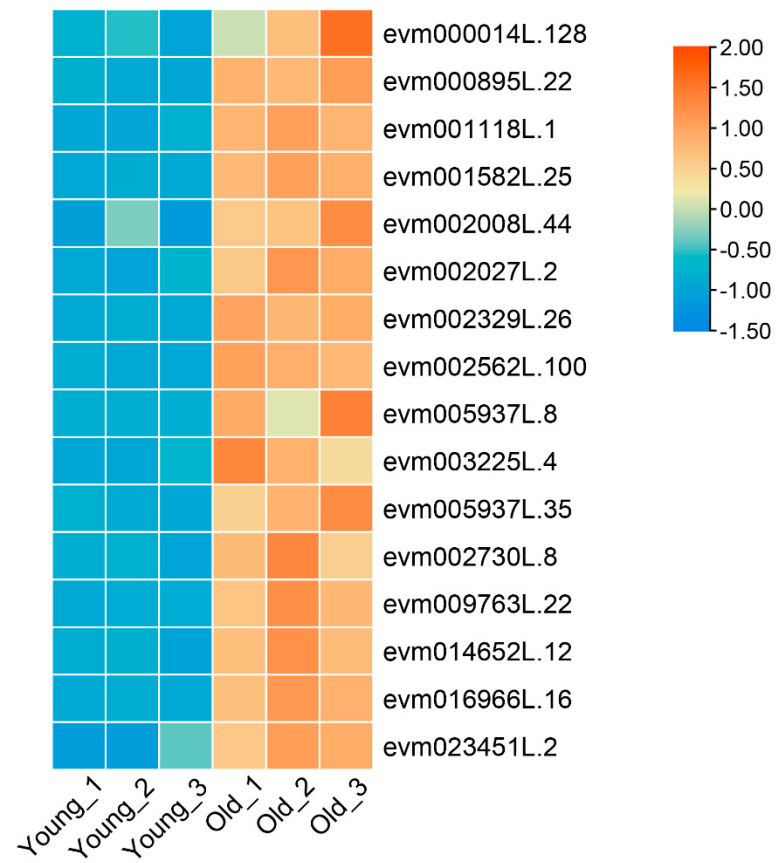

**Figure S1. A heatmap of DEGs related to the biosynthesis of key amino acids.**

Supplement: Supplementary file 1 [file ijms-24-17025-s001.zip › Supplemental Information.pdf]
